# Zika virus-based immunotherapy enhances long-term survival of rodents with brain tumors through upregulation of memory T-cells

**DOI:** 10.1371/journal.pone.0232858

**Published:** 2020-10-01

**Authors:** Andrew T. Crane, Matthew R. Chrostek, Venkatramana D. Krishna, Maple Shiao, Nikolas G. Toman, Clairice M. Pearce, Sarah K. Tran, Christopher J. Sipe, Winston Guo, Joseph P. Voth, Shivanshi Vaid, Hui Xie, Wei-Cheng Lu, Will Swanson, Andrew W. Grande, Mark R. Schleiss, Craig J. Bierle, Maxim C-J. Cheeran, Walter C. Low

**Affiliations:** 1 Department of Neurosurgery, University of Minnesota, Minneapolis, MN, United States of America; 2 Department of Veterinary Population Medicine, University of Minnesota, St. Paul, MN, United States of America; 3 Division of Pediatric Infectious Diseases and Immunology, Department of Pediatrics, University of Minnesota, Minneapolis, MN, United States of America; 4 Masonic Cancer Center, University of Minnesota, Minneapolis, MN, United States of America; University of Adelaide, AUSTRALIA

## Abstract

Zika virus (ZIKV) exhibits a tropism for brain tumor cells and has been used as an oncolytic virus to target brain tumors in mice with modest effects on extending median survival. Recent studies have highlighted the potential for combining virotherapy and immunotherapy to target cancer. We postulated that ZIKV could be used as an adjuvant to enhance the long-term survival of mice with malignant glioblastoma and generate memory T-cells capable of providing long-term immunity against cancer remission. To test this hypothesis mice bearing malignant intracranial GL261 tumors were subcutaneously vaccinated with irradiated GL261 cells previously infected with the ZIKV. Mice also received intracranial injections of live ZIKV, irradiation attenuated ZIKV, or irradiated GL261 cells previously infected with ZIKV. Long-term survivors were rechallenged with a second intracranial tumor to examine their immune response and look for the establishment of protective memory T-cells. Mice with subcutaneous vaccination plus intracranial irradiation attenuated ZIKV or intracranial irradiated GL261 cells previously infected with ZIKV exhibited the greatest extensions to overall survival. Flow cytometry analysis of immune cells within the brains of long-term surviving mice after tumor rechallenge revealed an increase in the number of T-cells, including CD4^+^ and tissue-resident effector/ effector memory CD4^+^ T-cells, in comparison to long-term survivors that were mock-rechallenged, and in comparison to naïve untreated mice challenged with intracranial gliomas. These results suggest that ZIKV can serve as an adjuvant to subcutaneous tumor vaccines that enhance long-term survival and generate protective tissue-resident memory CD4^+^ T-cells.

## Introduction

Glioblastoma multiforme (GBM) is a highly aggressive malignant brain tumor whose treatment options currently offer little chance of long-term survival or cure. Patients with GBM typically undergo surgical resection followed by treatment with chemotherapy and temozolomide [1], with a median overall survival (OS) of only 16 months [2]. Given the limitations of available therapies, new approaches to treat GBM are needed.

Oncolytic viruses offer a promising avenue for treating GBM. Viral tropism can be exploited to target tumor cells with greater efficiency and fewer side effects when compared to treatment with chemotherapy and temozolomide [3]. Recently, virotherapy has turned towards the immunogenic activity of viruses and their potential for immunotherapy [4]. Viruses are thought to induce an anti-tumor immune response through the direct lysis of infected cells and release of tumor-associated antigens, as well as the activation of antiviral pathways, creating an adaptive immune response against tumor and virus [5].

In the present study, we investigated Zika virus (ZIKV) as a virotherapy for GBM. ZIKV has been shown to exhibit a tropism for GBM [6, 7] and in mouse GBM models treatment with different viral strains, including a mouse-adapted Dakar ZIKV and a genetically modified Cambodian ZIKV, have improved OS [6, 8]. Here the therapeutic potential of the French-Polynesian (H/PF/2013) strain of ZIKV was assessed in the GL261mouse and 9L rat models of glioma. While ZIKV was capable of infecting GL261 cells *in vitro*, we observed no significant improvement in OS following intracranial (*i*.*c*.) injections of ZIKV in glioma-bearing mice or rats, unlike the survival improvements in a previous publication which used a mouse-adapted Dakar ZIKV to treat GL261-bearing mice [6]. An increase in pattern recognition receptor transcripts was observed following *in vitro* ZIKV infection of GL261 cells, however, which led us to interrogate ZIKV as an adjuvant to vaccine-based immunotherapy. It was observed that intratumoral treatment with a gamma-irradiated (IR), attenuated ZIKV (aZIKV) in combination with repeated vaccination of IR tumor cells previously infected with ZIKV significantly improves OS in GL261-mice. Additionally, we provide evidence of enhanced T-cell response in the brain of mice surviving long-term after tumor induction, specifically CD4^+^ and effector memory CD4^+^ T-cells, suggestive of long-term immunity against glioma.

## Methods

All methods involving the use of ZIKV and mice described here have been approved by the University of Minnesota Institutional Biosafety Committee (Protocol 1910-37492H) and the Institutional Animal Care and Use Committee (Protocol 1910-37491A). Any work involving ZIKV was performed under BSL2 containment.

### Cell culture

Mouse glioma cell line GL261-GFP.Luciferase (GL261; established and acquired from the lab of the late Dr. John Ohlfest [9]), rat glioma cell line GS-9L (9L; ECACC, 94110705), mouse microglia BV2 cell line ([10], acquired from the lab of Dr. Ling Li), and Vero cell line (African Green Monkey kidney epithelium; ATCC, CCL-81) were maintained with media changes every 48 hours and cells were passaged when reaching 80% confluence using TrypLE. Glioma media consisted of DMEM high glucose and L-glutamine (Genesee Scientific 25–500), supplemented with 10% Fetal Bovine Serum (Corning 35-011-CV), 1% Penicillin-Streptomycin (HyClone SV30010), 1% MEM NEAA (Gibco 1140–050). Vero media consisted of MEM Earle Salts supplemented with L-glutamine (Genesee Scientific 25–504), 10% Fetal Bovine Serum (Corning 35-011-CV), 1% Penicillin-Streptomycin (HyClone SV30010), 1% MEM NEAA (Gibco 1140–050).

### ZIKV

ZIKV H/PF/2013 (passage 4) was obtained from the European Virus Archive (001v-EVA1545) and cultured using previously established protocols [11]. ZIKV was passaged on Vero cells to generate working stocks of virus which were then concentrated by the ultracentrifugation of virus-containing media over a sucrose cushion, as described previously [12]. Multiple working ZIKV stocks were made from the same parent stock. All working stocks were aliquoted and stored at -80°C. Working stock from the same preparation was used across all groups in individual experiments.

ZIKV titers were calculated by titration and plaque assay [13]. Briefly, 3 x 10^5^ Vero cells were plated in each well of a 6-well plate the day before infection and allowed to form a monolayer. The day after creating plates, 10-fold serial dilutions of ZIKV (10^−1^ to 10^−6^ in 1 mL Vero media) was prepared in triplicate and was placed on each well and allowed to adsorb for 2 hours. After the adsorption period, a PBS wash was conducted to remove remaining virus not adsorbed, and finally a solution of 1.5mL of 2x concentrated Vero media and 1.5mL of 1.1% SeaPlaque low-melting agarose at 37°C was applied over the monolayers. This mixture was allowed to cool to room temperature, forming a gelatinous overlay. After four days, 4% PFA was applied for a minimum of 2 hours to fix the virus, cells, and overlays. The overlays were removed by applying warm tap water and manually tapping the plate, and 0.1% crystal violet was used to stain the cells and easily identify plaques. Plaques were counted under a dissection microscope and the concentration of virus in each day’s supernatant was calculated. Averages of technical triplicates were used to calculate the concentration of virus.

For *in vivo* infection, ZIKV was diluted in PBS to achieve the desired infectious dose. To make aZIKV, the desired concentration of ZIKV was irradiated in-house at 60 Gy for 20 minutes with gamma irradiation from a Cs-137 irradiator. This dose of irradiation is not sufficient to inactivate the virus as plaques were present in aZIKV infected Vero cells using a plaque assay.

### ZIKV infection of GL261 cells

To determine the infectivity of ZIKV in GL261 mouse glioma cells, biological triplicates of 2–3 x 10^6^ cells were infected with ZIKV at an MOI of 0.01 for two hours. Following incubation, the virus containing media was removed, the cells were rinsed with PBS to remove virus not adsorbed, and 4mL of fresh non-virus containing media was placed on the cells. Following the infection, virus-containing media was collected at 1-, 2-, and 3-days post-infection (DPI) and stored at -80°C. Average concentration of viral titers from biological triplicate, at each time point was quantified using the plaque assay described above.

### RNA isolation

For RNA isolation, 2–3 x 10^6^ cells (GL261 and BV2) were infected with ZIKV at an MOI of 0.01 for two hours. Virus containing media was removed, cells were washed with PBS to remove any residual virus, and fresh media was added. Every 24 hours following infection, for three days, cells were lysed in RLT buffer and stored at -80°C before RNA isolation. Control samples of uninfected GL261 and BV2 cells were also collected. RNA was isolated from infected (1-DPI and 3-DPI) and uninfected GL261 and BV2 cells using the Qiagen RNeasy Plus Mini Kit following manufacturers’ instructions. Cell counts were performed in ZIKV-infected and uninfected cells and RNA was normalized to cell counts post-extraction.

### cDNA synthesis and qRT-PCR

cDNA was synthesized from 500 ng of each respective RNA sample using the ProtoScript® First Strand cDNA Synthesis Kit (New England BioLabs, E6300L) according to manufacturer’s instructions.

Using the Eppendorf Realplex 2 PCR system, qRT-PCR was performed using 100 ng of cDNA with 2.5 μl of specific primers, targeting MDA5, RIG-I, STAT2, IFN-β, TLR3 (S1 Table), and 12.5 μl of Apex qPCR 2X GREEN Master Mix (Apex Bioresearch Products) in a final reaction volume of 25 μl. The cycling conditions for MDA5, RIG-I, and STAT2 were 40 cycles of 95°C for 30 s, 55°C for 40 s, and 72°C for 30 s. The cycling conditions for IFN-β and TLR3 were 40 cycles of 95°C for 30 s, 50°C for 40 s, and 72°C for 30 s. mRNA expression fold change (over uninfected cells) was quantified by calculating 2^-ΔΔCT^ with HPRT mRNA as an endogenous control.

### Tumor vaccine

Individual mouse or rat tumor vaccine products were generated using the mouse GL261 glioma cell line or rat 9L glioma cell line, respectively. Vaccines consisted of IR glioma cells previously infected with ZIKV, coupled with GM-CSF. Briefly, cells were incubated with 0.5 MOI ZIKV for 48 hours, after which the media was removed and replaced with fresh media. The cells were cultured for an additional 48 hours followed by dissociating the culture monolayer with TrypLE and to obtain a cell pellet. The pellet was resuspended in PBS and, using an in-house Cs-137 irradiator, irradiated with gamma rays for 60 Gy for 20 minutes at room temperature. The irradiated cell solution was centrifuged, then resuspended in PBS and total cell number was counted using a hemocytometer. The cells were centrifuged and resuspended in freezing media (glioma media with 10% DMSO) at a concentration of 1 x 10^7^ cells/mL/cryovial. The cryovial was frozen in isopropanol chambers at -80°C until use. On the days in which animals were given vaccine, cryovials were removed from -80°C, thawed in a 37°C water bath, and then resuspended in warm glioma media. Cells were centrifuged, washed in glioma media followed by another centrifugation. The resulting cell pellet was then resuspended in 1mL PBS with 20ng GM-CSF. Mice or rats in the vaccination groups received a subcutaneous injection of 500 μL.

### Mouse tumor studies

#### Preparation of GL261 for tumor induction

Prior to surgery, GL261 cells were prepared for transplantation by washing three times with PBS, then trypsinized for 5 minutes at 37°C. The cell suspension was collected then centrifuged and the resulting pellet was resuspended in cold DMEM for counting viable cells using the Trypan Blue exclusion method. The cells were centrifuged a second time and resuspended at a concentration of 1 x 10^4^ cells per μL in cold DMEM and placed on ice.

#### Tumor induction and treatment

10-week old C57BL/6J and NOD-SCID mice were purchased from Jackson Laboratories. Mice were first anesthetized with isoflurane oxygen mixture, then the head of the animal was shaved and treated with betadine. Following mounting in a stereotaxic frame, a single midline incision was made along the scalp and skin retracted to expose bregma. A small burr hole was drilled in the skull above the injection site in the right hemisphere (from bregma: anterior 1.0 mm and lateral 1.5 mm). A 10 μL Hamilton syringe loaded with the cell solution was slowly inserted into the brain (3.1 mm ventral to the pia mater in mice) and 1 x 10^4^ viable cells in 1 μL were injected at a rate of 0.5 μL/minute. Following injection, the needle remained in place for one minute. The injection needle was raised 0.1 mm then 0.2 mm from the initial injection site and the injection was repeated with 1 x 10^4^ cells injected at each site for a total of 3 x 10^4^ viable cells (total volume of 3 μL). At the conclusion of the last injection, the needle remained in place for three minutes before being slowly withdrawn.

Mice scheduled for treatment with *i*.*c*. ZIKV or aZIKV immediately following tumor induction remained in the stereotaxic frame. A 10 μL Hamilton syringe was loaded with 5 x 10^4^, 5 x 10^6^, or 5 x 10^8^ pfu/μL of ZIKV or 5 x 10^4^ pfu/μL aZIKV. The needle was slowly inserted into the brain (3.0 mm) and 1 μL of virus was injected at a rate of 1 μL/minute. At the conclusion of the injection, the needle remained in place for one minute before being slowly withdrawn. Mice not treated with *i*.*c*. ZIKV were injected with sterile saline at the same coordinates. At the conclusion of the surgery, the incision site was cleaned and closed using a 7mm Reflex wound stapler. Mice were transferred to a heated recovery cage until fully sternal, then returned to their homecage. In the first mouse tumor study, on days 3, 7, and 14 following tumor-induction, mice in the vaccination groups received a subcutaneous injection of mouse vaccine (500 μL) between the shoulder blades. In the second mouse tumor study, mice in the vaccination groups received injections on days 3, 7, and 21.

Mice scheduled for treatment with *i*.*c*. aZIKV or *i*.*c*. vaccine 14-days post-tumor induction were anesthetized and prepped for surgery as described above with the center of the burr hole from the prior surgery serving as the injection site. The needle was slowly inserted into the brain (3.25 mm ventral to the pia mater in mice) and 1 μL, containing 5 x 10^4^ pfu/μL aZIKV or 3 x 10^4^ IR GL261 cells previously infected with ZIKV, was injected at a speed of 0.5 μL/minute. Following injection, the needle remained in place for one minute. The injection needle was raised 0.75 mm from the initial injection site and the injection was repeated, totaling 1 x 10^5^ pfu aZIKV or 6 x 10^4^ IR GL261 cells previously infected with ZIKV. At the conclusion of the last injection, the needle remained in place for three minutes before being slowly withdrawn. Mice were transferred to a heated recovery cage until fully sternal, then returned to their homecage.

#### Tumor rechallenge

Long-term survivors (118-days and 86-days following initial tumor induction for the first and second mouse tumor study, respectively) were injected with 3 x 10^4^ GL261 cells in both the left- and right-hemispheres, following the procedure described above. Seven days following rechallenge, mice were deeply anesthetized with an overdose of Ketamine (100mg/kg) followed by transcardial perfusion with ice-cold PBS. The brain was removed and the right hemisphere was extracted and dissociated for flow cytometry. Cervical lymph nodes from mice in the second study were extracted for IFNγ ELISA. Control groups included long-term surviving mice with an *i*.*c*. injection of saline, age-matched naïve controls with *i*.*c*. tumor, and age-matched naïve controls with *i*.*c*. saline injection.

#### Flow cytometry

Mononuclear cells isolated from tumor rechallenged mouse brains were isolated by a density gradient, as previously described [14]. Briefly, brains were finely minced using razor blades, mechanically disrupted by pipetting up and down, and suspended in RPMI medium with 30% Percoll. This suspension was transferred to a 15 ml conical tube and slowly underlayed with 1.5 ml of 70% Percoll solution. After centrifugation at 900 x g for 30 min at 15°C, the mononuclear cells in the interphase were collected and washed twice with PBS. Cells were stained with LIVE/DEAD^TM^ fixable Aqua dead cell stain (Thermo Fisher Scientific, Rockford, IL) for 30 min at 4°C, blocked with anti-mouse CD16/CD32 (Fc block, BD Biosciences) for 5 min at RT, and stained with following anti-mouse immune cell markers for 30 min at 4°C. CD45-PerCP-Cyanine5.5, CD11b-APC-eFluor 780, Ly6c-eFluor 450, CD86-FITC, MHC class II-PE, CD19-PE, CD3e-eFluor 450, CD4-PE-Cy7, CD8-FITC, CD44-APC, CD25-PE, (eBioscience, San Diego, CA), CD11c-Brilliant Violet 711, Ly6G-PE-Cy7, CD206-Alexa Fluor 647, CD62L-Brilliant violet 605, NK1.1-Brilliant violet 650, CD69-Brilliant violet 711, (BioLegend, San Diego, CA). To identify Tregs, surface stained cells were fixed, permeabilized and stained with FoxP3-APC using anti-mouse/rat FoxP3 staining set (eBioscience, San Diego, CA). Isotype specific antibodies were used to control for nonspecific antibody binding. Immunostained cells were acquired using BD LSRFortessa X-20 flow cytometer (BD Biosciences) and data were analyzed using FlowJo software. SPHERO AccuCount particles (Spherotech, Lake Forest, IL) were added to samples immediately before analysis to calculate absolute number of each cell population.

#### *In vitro* stimulation and IFN-γ detection

Cervical lymph nodes (CLN), from rechallenged long-term surviving mice in the second mouse tumor study were mechanically disrupted to prepare single-cell suspension and passed through 70 μm cell strainer. Cells were washed twice with PBS and resuspended in complete RPMI medium (RPMI 1640 medium supplemented with 10% FBS, 2mM L-glutamine, 1mM sodium pyruvate, 20mM HEPES, and 1x Penicillin-Streptomycin). For IFN-γ assay, 0.5x10^6^ live cells were seeded in 200 μL per well in a 96-well flat bottom tissue culture plate and stimulated in triplicate wells with IR GL261 cell lysate (50μg/mL) or heat-inactivated ZIKV (3.5x10^6^ pfu/mL). Cells stimulated with Concanavalin A (10 μg/mL) or unstimulated cells were included as positive and negative controls respectively. Cells were incubated at 37°C/5% CO_2_ for 72 h. Culture supernatants were collected at 72 h of stimulation and concentration of interferon-γ (IFN-γ) was determined using mouse IFN-gamma DuoSet ELISA (DY485, R & D systems, Minneapolis, MN) according to manufacturer’s instructions.

### Rat tumor induction and treatment

11-week old F344 rats weighing 225-250g were purchased from Taconic. Animals were first anesthetized with isoflurane oxygen mixture, then the head of the animal was shaved and treated with betadine. Following mounting in a stereotaxic frame, a single midline incision was made along the scalp and skin retracted to expose bregma. A 10 μL Hamilton syringe was loaded with the 9L cell solution. 9L cells were prepared for tumor induction following methods outlined in “Preparation of GL261 for tumor induction”. A small burr hole was drilled in the skull above the injection site in the right hemisphere (from bregma: anterior 1.0 mm and lateral 3.0mm). The needle was slowly inserted into the brain (5 mm ventral to pia mater in rats) and 2.5 x 10^4^ viable cells were injected at a speed of 0.5 μL/minute. Following injection, the needle remained in place for one minute. The injection needle was raised 0.1 mm from the initial injection site and the injection was repeated with 2.5 x 10^4^ cells injected at each site for a total of 5 x 10^4^ viable cells. At the conclusion of the last injection the needle remained in place for three minutes before being slowly withdrawn. Animals scheduled for treatment with *i*.*c*. ZIKV were transferred to a BSL-2 laminar flow hood while remaining under isoflurane anesthesia in the stereotaxic frame. A 10 μL Hamilton syringe was loaded with ZIKV or aZIKV at viral titers of 5 x 10^4^ pfu/μL. The needle was slowly inserted into the brain (4.5 mm) and 1 μL of virus was injected at a speed of 1 μL/minute. At the conclusion of the injection the needle remained in place for one minute before being slowly withdrawn. Animals not treated with ZIKV were injected with *i*.*c*. sterile saline. At the conclusion of the surgery, the incision site was cleaned and closed using a 9mm Reflex wound stapler. Animals were transferred to a heated recovery cage until fully sternal. On days 3, 7, and 14 following tumor-induction, animals in the vaccination groups received a subcutaneous injection of rat vaccine (500 μL) between the shoulder blades.

### Data analysis and visualization

Survival data was analyzed using RStudio Environment (Version 1.0.153) using the Chi-Squared Kaplan-Meier and Long-Rank methods in the Survival package. Pairwise comparisons of each treatment group against untreated tumor-bearing animals in each study was corrected using Bonferroni correction for multiple comparisons. Flow cytometry analysis and IFN-γ detection statistical analysis was performed by one-way analysis of variance (ANOVA) with Tukey post hoc correction for multiple comparisons using GraphPad Prism 8 (GraphPad Software, San Diego, CA). An α level < 0.05 is considered statistically significant unless otherwise noted.

## Results

### GL261 cells are permissive to ZIKV infection

**Fig 1:**
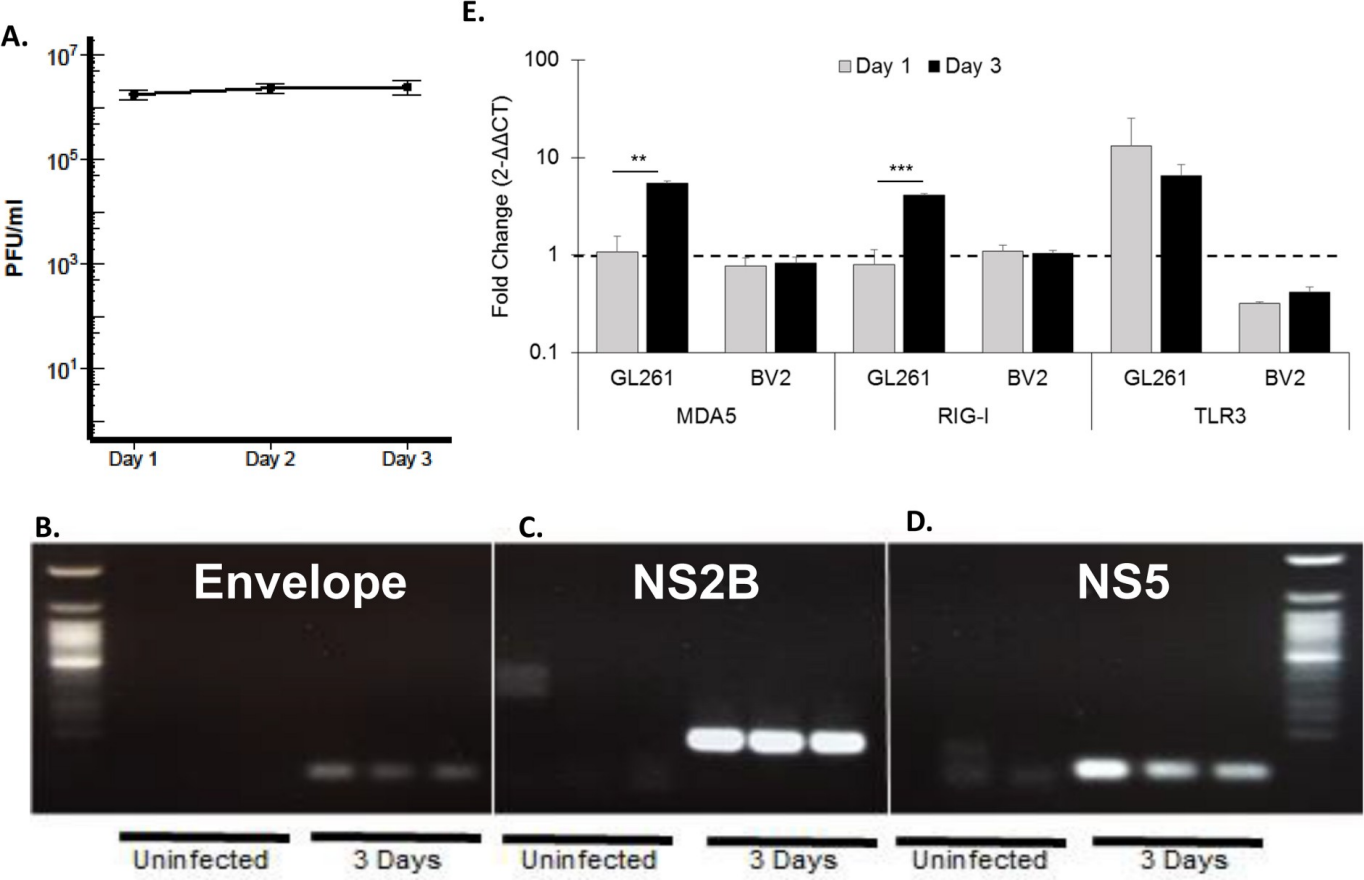
*In vitro* ZIKV infection of the murine GL261 glioma cell line. (A) Plaque forming assay demonstrating permissiveness of ZIKV to infect and release virus, measured at 24-hour intervals following initial infection of 0.01 MOI (~2–3 x 10^4^ PFU). PCR and electrophoresis-based detection of ZIKV transcripts for Envelope (B), NS2B (C), and NS5 (D) 3-days following infection in GL261 cells. (E) Expression of viral pattern recognition receptor (MDA5, RIG-I, and TLR3), in ZIKV infected cells at 1- and 3- days post-ZIKV infection, measured via qRT-PCR. Data represent mean ± SEM of biological triplicates (n = 3 per time point). (*p < 0.05; **p < 0.01; ***p < 0.001). Data was acquired from single independent experiments.

The ability for ZIKV to infect, replicate and release virus in GL261 cells was measured by plaque assay on Vero cells. An increase in virus particles within the media of infected cells was observed at one day post-infection (DPI) which remained elevated over time (Fig 1A). RNA isolated from lysed GL261 cells 3-DPI further confirmed the ability for ZIKV to infect GL261 cells as transcripts for the envelope (Fig 1B), non-structural 2 subunit (Fig 1C), and non-structure 5 subunit (Fig 1D) were observed in the infected samples. Finally, we assessed antiviral response gene expression following ZIKV infection in GL261 cells and a ZIKV non-permissive mouse microglia (BV2) [6]. Lysates from GL261 and BV2 cell lines were collected at 1-DPI and 3-DPI for qRT-PCR analysis of transcripts related to endosomal (TLR3) and cytoplasmic (MDA5 and RIG-I) viral RNA detection [15]. Relative to mock-infected cells, an increase in expression of TLR3 was observed at 1-DPI and 3-DPI, and increases in expression of MDA5 and RIG-I were observed at 3-DPI, which was not observed in BV2 cells (Fig 1E). These data suggest that, similar to previous studies, GL261 cells are permissive to ZIKV infection and propagation *in vitro*.

### Oncolytic effects of ZIKV in experimental glioma

**Fig 2:**
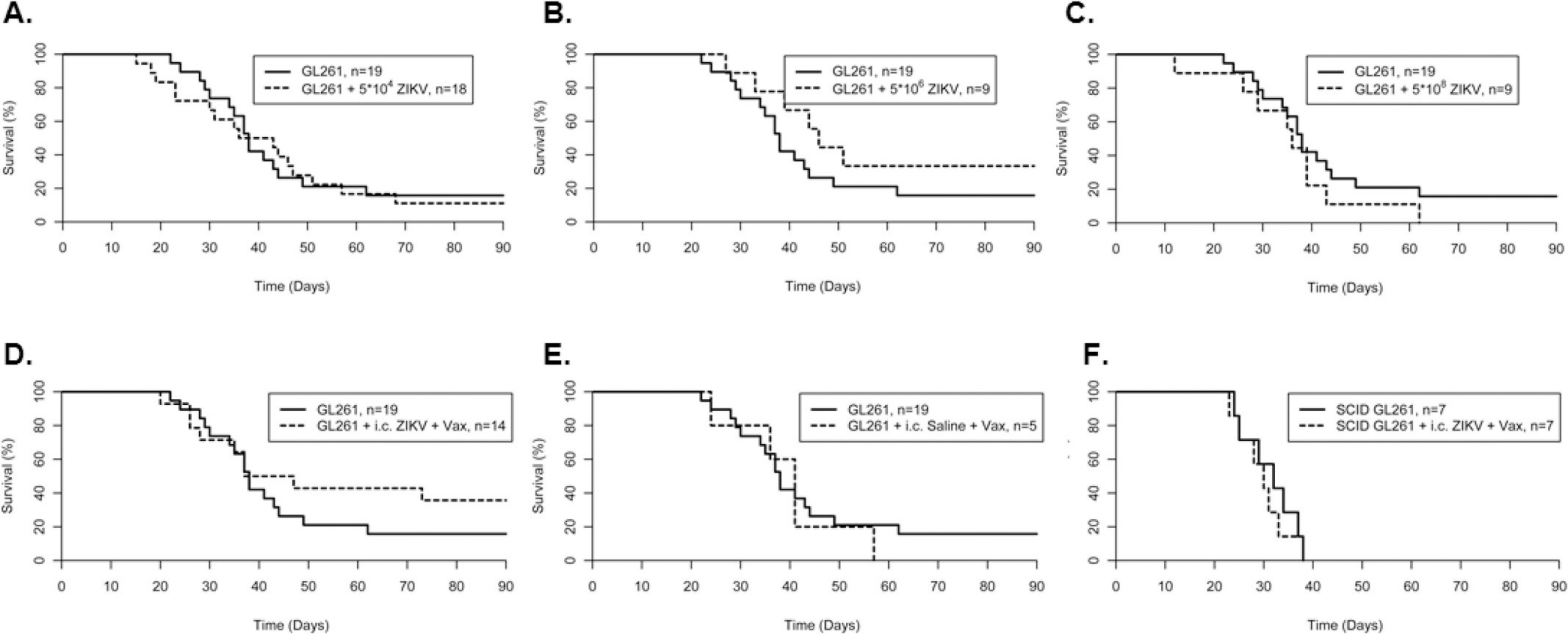
Survival plots of first mouse tumor study. C57BL6/J mice implanted with murine GL261 glioma cells treated with intracranial (*i*.*c*.) injection of ZIKV at the site of the tumor at (A) 5x10^4^, (B) 5x10^6^, (C) 5x10^8^ pfu, (D) *i*.*c*. injection of 5x10^4^ pfu ZIKV coupled with subcutaneous vaccination of IR GL261 cells previously infected with ZIKV (GL261 + i.c. ZIKV + Vax), or (E) subcutaneous vaccination of IR GL261 cells (GL261 + i.c. saline + Vax) all relative to untreated GL261 implanted mice. (F) NOD/SCID mice implanted with murine GL261 glioma cells treated with *i*.*c*. injection of 5x10^4^ ZIKV coupled with subcutaneous vaccination of irradiated GL261 cells previously infected with ZIKV (SCID GL261 + i.c. ZIKV + Vax) compared to untreated GL261 implanted NOD/SCID mice. Data in (A-E) was acquired and combined from multiple experiments. Data in (F) was acquired from a single independent experiment.

To assess the ability of ZIKV to improve OS, *in vivo*, immunocompetent C57BL/6J mice were implanted with a single-cell suspension of 3 x 10^4^ GL261 cells directly into the striatum followed immediately by *i*.*c*. injections of 5 x 10^4^, 5 x 10^6^, or 5 x 10^8^ pfu ZIKV at the same coordinates. Mice were monitored daily for a moribund state as endpoint criteria for overall survival (OS) up to 118 days post-tumor induction. No significant alteration in OS in any of the ZIKV treated GL261-mice was observed (Fig 2A–2C). In untreated GL261-mice, a median survival of 38 days with an OS of 15.8% was observed (Table 1). Only in GL261-mice treated with the middle dose of ZIKV (5x10^6^ pfu) was a non-significant trend in median survival of 46 days and an OS of 33.3% observed.

**Table 1:** Descriptive statistics of first mouse tumor study.

Group	n	Median Survival (days)	Overall Survival (%)	Pairwise *p*
GL261	19	38	15.8	---
GL261 + *i*.*c*. 5x10^4^ ZIKV	18	39.5	11.1	0.85^*a*^
GL261 + *i*.*c*. 5x10^6^ ZIKV	9	46	33.3	0.21^*a*^
GL261 + *i*.*c*. 5x10^8^ ZIKV	9	36	0	0.29^*a*^
GL261 + *i*.*c*. 5x10^4^ ZIKV + Vax	14	42	35.8	0.32^*a*^
GL261 + *i*.*c*. saline + Vax	5	41	0	0.68^*a*^
SCID GL261	7	32	0	---
SCID GL261 + *i*.*c*. 5x10^4^ ZIKV + Vax	7	30	0	0.61

Summary of descriptive statistics in first mouse tumor study. Data of GL261 in C57BL6/J mice were compiled from multiple independent experiments. Data of GL261 in SCID mice were from a single independent experiment.

^a^ Pairwise Log-Rank tests of each treatment group against untreated GL261 mice was corrected using Bonferroni method for multiple comparisons. *p* < 0.01 is considered statistically significant.

**Fig 3:**
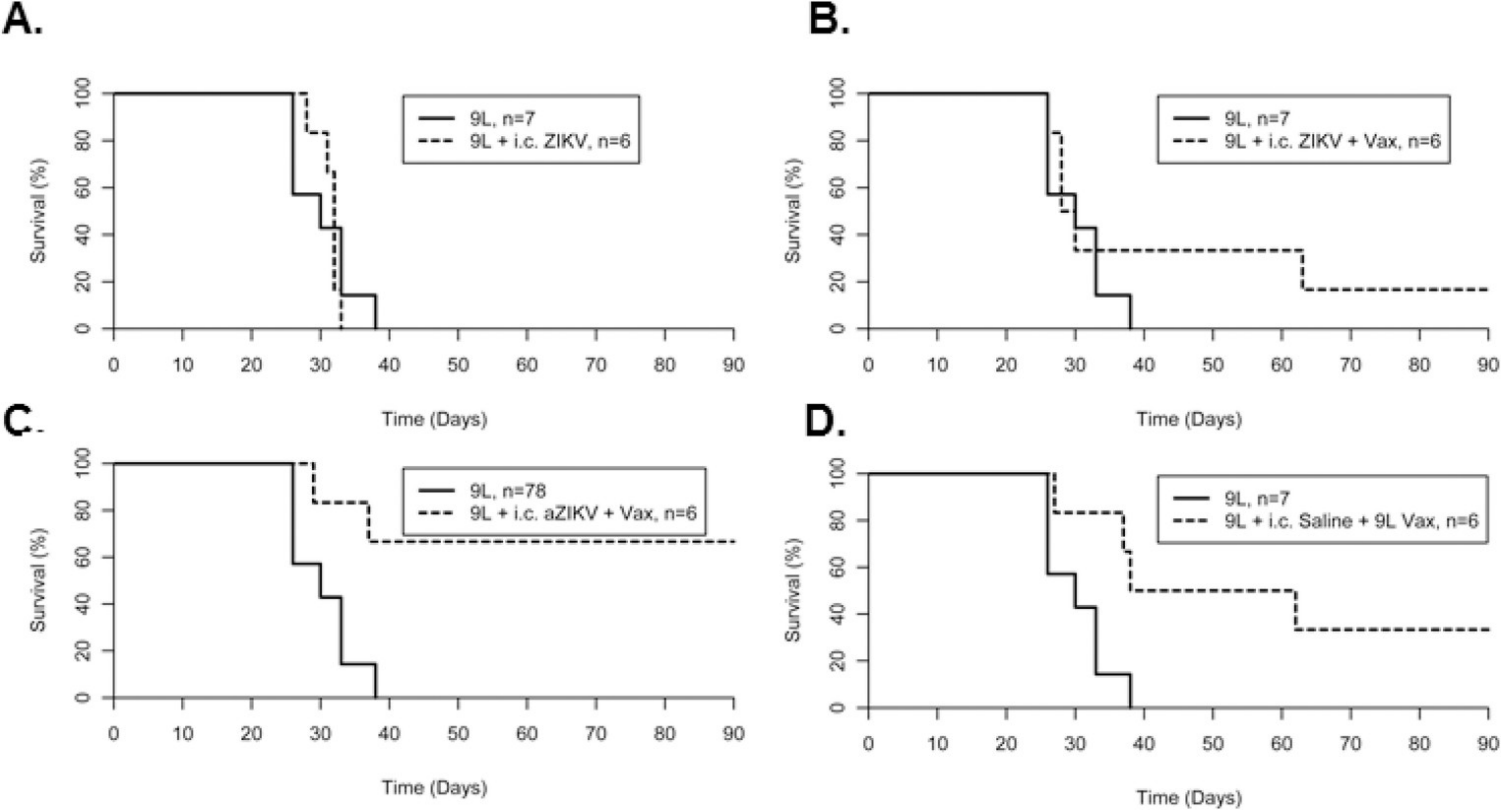
Survival plots of rat tumor study. F344 rats implanted with rat 9L glioma cells treated with either (A) intracranial (*i*.*c*.) injection of 5x10^4^ ZIKV (9L + i.c. ZIKV), (B) *i*.*c*. injection of 5x10^4^ ZIKV coupled with subcutaneous vaccination of IR 9L cells previously infected with ZIKV (9L + i.c. ZIKV + Vax), (C) *i*.*c*. injection of 5x10^4^ aZIKV coupled with subcutaneous vaccination of irradiated 9L cells previously infected with ZIKV (9L + i.c. aZIKV + Vax), or (D) subcutaneous vaccination of irradiated 9L cells that have not been infected with ZIKV (9L + i.c. saline + 9L Vax) compared to untreated 9L implanted rats. Data was acquired from a single independent experiment.

Similarly, we tested the oncolytic effect of ZIKV in a rat glioma model, where 5 x 10^4^ rat 9L cells were transplanted into in F344 rats and immediately treated with an *i*.*c*. injection of 5 x 10^4^ live ZIKV at the same coordinates. Treatment of rats with *i*.*c*. injection of ZIKV alone did not improve OS (median survival of 32 days and OS of 0%) relative to untreated 9L tumor-bearing rats (median survival of 30 days and OS of 0%; Fig 3A and Table 2). These data indicate that ZIKV alone is not sufficient as a therapy for glioma.

**Table 2:** Descriptive statistics of rat tumor study.

Group	n	Median Survival (days)	Overall Survival (%)	Pairwise *p*^*a*^
9L	7	30	0	---
9L + *i*.*c*. 5x10^4^ ZIKV	6	32	0	0.78
9L + *i*.*c*. 5x10^4^ ZIKV + Vax	6	29	16.7	0.38
9L + *i*.*c*. 5x10^4^ aZIKV + Vax	6	n/a	66.7	0.01*
9L + *i*.*c*. saline + 9L Vax	6	50	33.3	0.02

Summary of descriptive statistics in rat tumor study. Data were from a single independent experiment.

^a^ Pairwise Log-Rank tests of each treatment group against untreated 9L rats was corrected using Bonferroni method for multiple comparisons. *p* < 0.0125 is considered statistically significant.

### ZIKV & tumor vaccine co-therapy in experimental glioma

Given that ZIKV was ineffective alone, we hypothesized that it could instead serve as an adjuvant to enhance the anti-tumor effects of a vaccine-based therapy. To test this, GL261-mice were treated with *i*.*c*. injections of ZIKV as well as subcutaneous vaccination (comprised of 5x10^6^ IR GL261 cells previously infected with ZIKV and 20ng of the dendritic cell adjuvant GM-CSF) on days 3, 7, and 14 post-tumor induction. OS of GL261-mice in the *i*.*c*. ZIKV plus vaccine-treated group was at 35.8% compared to 15.8% in the untreated controls and 0% in GL261-mice treated only with vaccines (Fig 2D and 2E and Table 1). Pairwise Kaplan-Meier analysis with Bonferroni correction, however, did not reach significance. In the 9L model of glioma, rats treated with ZIKV coupled with a 9L-based vaccine had a non-significant increase in OS (Fig 3B and Table 2). Interestingly, a significant increase in OS was observed in 9L bearing rats treated with the combination of *i*.*c*. aZIKV and subcutaneous vaccines (OS of 66.7%; pairwise *𝜒*^2^ *=* 6.5, *p* = 0.011; Fig 3C and Table 2), although it is currently not clear the mechanism by which attenuation of ZIKV by IR exerts a beneficial effect.

To evaluate the role of the immune system in this therapy, immunodeficient NOD-SCID mice were implanted with GL261 cells and subjected to the same ZIKV and vaccine treatment. Relative to untreated NOD-SCID GL261-mice, no significant difference in OS was observed (Fig 2F and Table 1). Although it was not found to be statistically significant, an increase in the percent of immunocompetent treated mice surviving long term, relative to untreated mice, was not observed in the immunodeficient treated mice. This may suggest a possible mechanism in which *i*.*c*. ZIKV administration coupled with subcutaneous vaccination generates an adaptive immune response.

**Fig 4:**
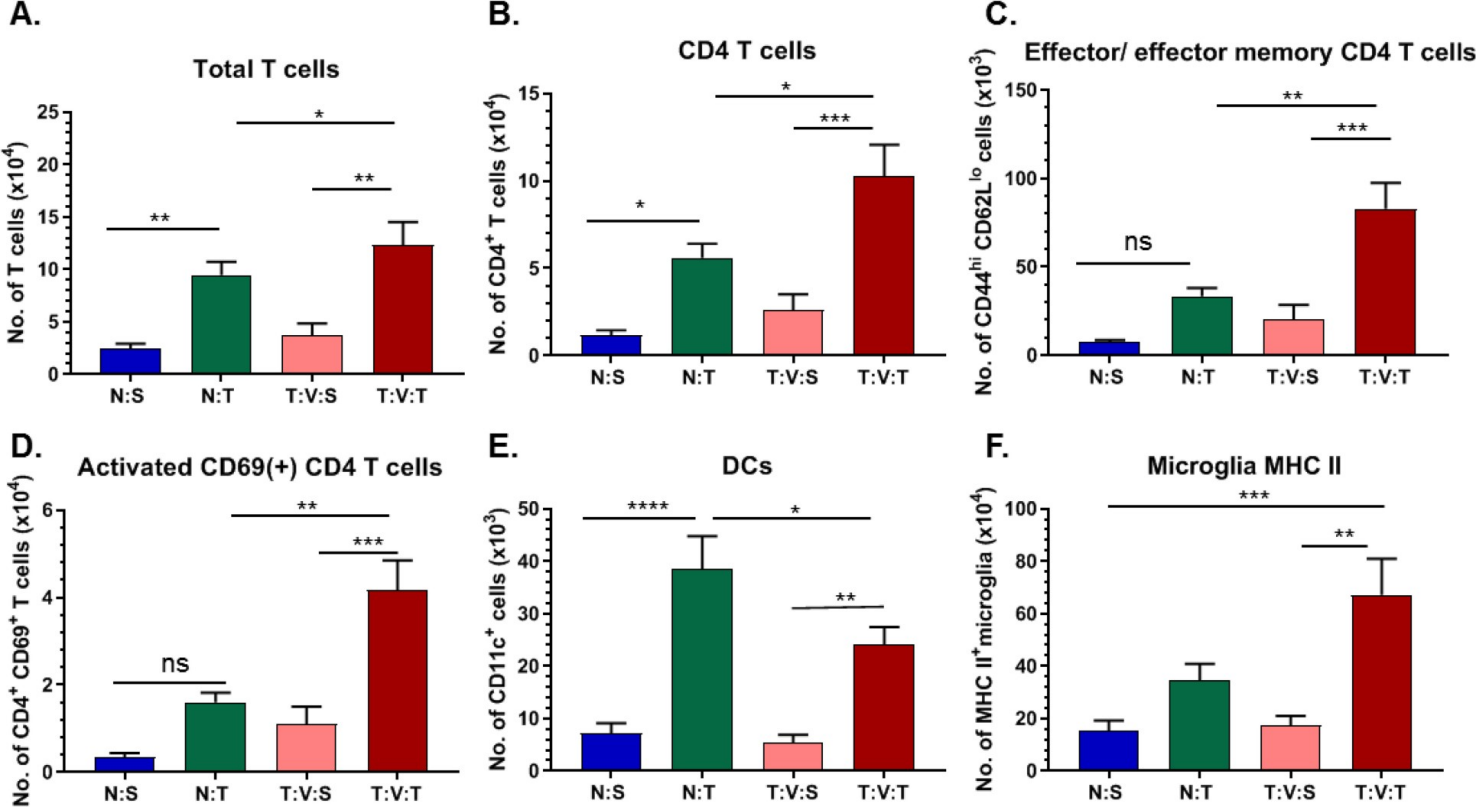
Phenotype of infiltrating and resident immune cells in tumor rechallenged mice. Flow cytometry analysis of mononuclear cells within the brains of long-term survivors of first vaccine treatment study 7 days following tumor rechallenge (T:V:T, *n* = 5), long-term survivors 7 days following intracranial (*i*.*c*.) injection of saline (T:V:S, *n* = 4), age-matched naïve-mice 7 days following *i*.*c*. tumor injection (N:T, *n* = 8), or age-matched naïve-mice 7 days following *i*.*c*. saline injection (N:S, *n* = 4). Number of (A) CD3^+^ T-cells, (B) CD4^+^ T-cells, (C) CD4+ CD44^hi^ CD62L^lo^ effector and effector memory T-cell, (D) activated CD4^+^ CD69^+^ T-cells (E) dendritic cells, and (F) activated MHC II^+^ microglia. Data represent mean ± SEM, (*p < 0.05; **p < 0.01; ***p < 0.001). Data was acquired from a single independent experiment.

To further investigate the role of the immune system in this adjuvant vaccine treatment paradigm, long-term survivors of the *i*.*c*. ZIKV plus vaccine-treated group were subjected to a second injection of GL261 cells approximately 114 days post-tumor-induction. Long-term survivors and age-matched naïve controls were implanted with either *i*.*c*. 3 x 10^4^ GL261 cells or *i*.*c*. saline at the same coordinates. Seven days following rechallenge, brains were collected for flow cytometry (S1 Fig). Relative to saline-injected long-term survivors and age-matched naive tumor-implanted mice, we observed significant increases in the number of total T-cells (CD3^+^; Fig 4A) and CD4^+^ T-cells (Fig 4B) in tumor-rechallenged long-term survivors. CD4^+^ effector T-cells and effector memory T-cells (Fig 4C) and CD69^+^ activated CD4^+^ T-cells (Fig 4D) were similarly increased in rechallenged long-term survivors. Increases in MHCII(hi)/CD11c^+^ dendritic cells and activated MHC II^+^ microglia were also observed in tumor-rechallenged long-term survivors relative to saline-injected long-term survivors (Fig 4E and 4F). These results reflect a coordinated activation of immune cells in long-term survivors, suggesting that *i*.*c*. ZIKV and tumor vaccine immunotherapy enhances a T-cell response that can be observed in a recurring tumor model.

### Irradiated ZIKV and tumor vaccine immunotherapy in experimental glioma

To increase the efficacy of the vaccine therapy, we sought to treat glioma bearing mice with *i*.*c*. injections of our therapies after a sufficient T-cell response has been generated by peripheral vaccination. It has been reported that vaccinations in mice are expected to yield a peak T-cell proliferation approximately 10 days after the first vaccine injection [16]. We therefore interrogated the effect of *i*.*c*. injections of our treatments on day 14, when T-cell proliferation should be near the peak response. We also tested *i*.*c*. injection of IR GL261 cells previously infected with ZIKV to amplify available tumor antigen at the site of the tumor.

In this second treatment schedule, GL261-mice were administered subcutaneous vaccines on days 3, 7, and 21. In addition to subcutaneous vaccines, one group received *i*.*c*. injections of 5 x 10^4^ aZIKV immediately following tumor induction (day 0) and a second group on day 14. A third group received *i*.*c*. injection of IR GL261 cells previously infected ZIKV (*i*.*c*. vaccine) on day 14.

**Fig 5:**
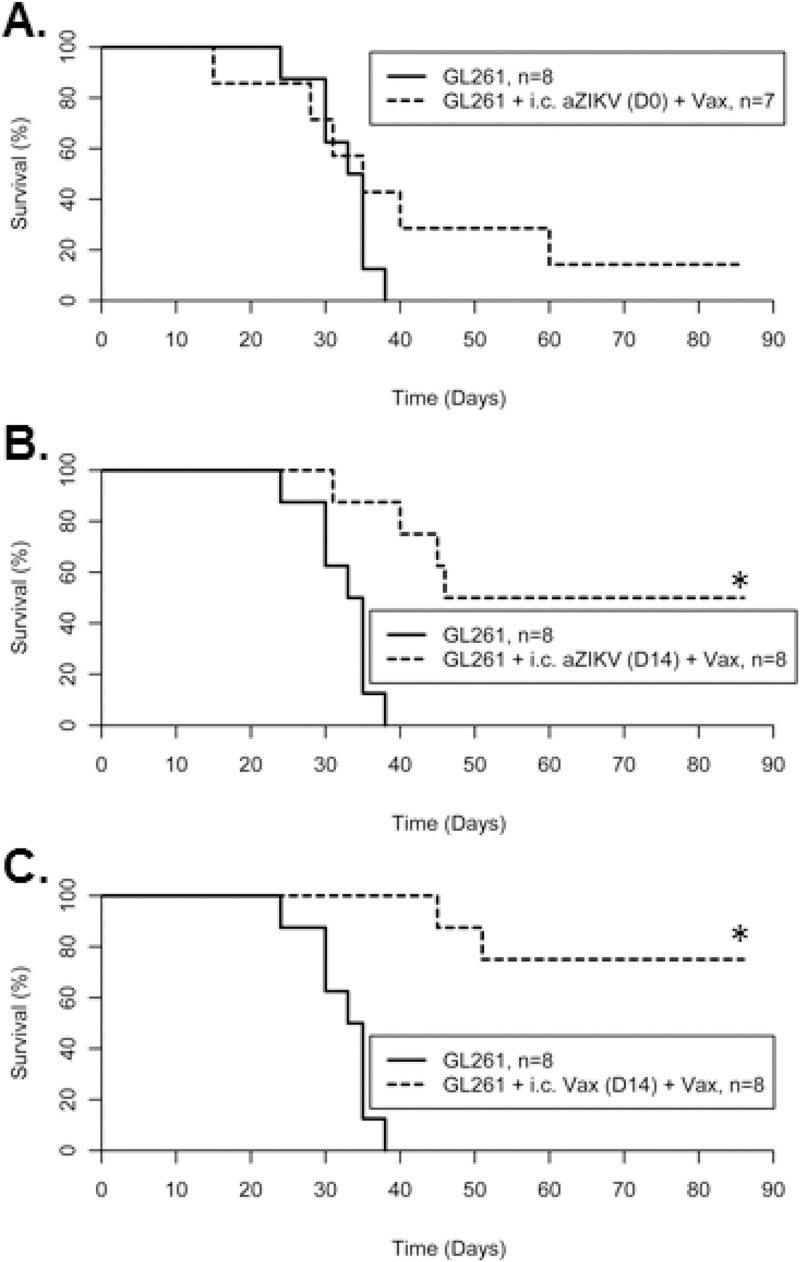
Survival plots of second mouse vaccine study. C57BL6/J mice implanted with murine GL261 glioma cells treated with (A) intracranial (*i*.*c*.*)* injection of 5x10^4^ pfu irradiated ZIKV at Day 0, (B) *i*.*c*. injection of 1x10^5^ pfu irradiated ZIKV at Day 14, or (C) *i*.*c*. injection of 6x10^4^ irradiated GL261 cells previously infected with ZIKV (i.c. Vax) at Day 14 all relative to untreated GL261 implanted mice. All treated mice were injected with subcutaneous vaccines on days 3, 7, and 21. Data was acquired from a single independent experiment.

Similar to the first treatment schedule, subcutaneous vaccinations of GL261-mice coupled with *i*.*c*. aZIKV immediately following tumor induction did not significantly improve median survival (35 days) or OS (14.3%), relative to untreated GL261-mice (Fig 5A and Table 3). Delaying *i*.*c*. aZIKV injection until day 14 significantly increased OS, relative to untreated mice (50%; pairwise *𝜒*^2^ *=* 11.1, *p* < 0.001; Fig 5B). Furthermore, treating GL261-mice with subcutaneous vaccinations coupled with *i*.*c*. vaccine on day 14 resulted in the greatest number of mice surviving long-term (75% OS; pairwise *𝜒*^2^ *=* 16.1, *p* < 0.001; Fig 5C).

**Table 3:** Descriptive statistics of second mouse tumor study.

Group	n	Median Survival (days)	Overall Survival (%)	Pairwise *P*^*a*^
GL261	8	34	0	---
GL261 + Vax + *i*.*c*. 5 x 10^4^ aZIKV (D0)	7	35	14.3	0.23
GL261 + Vax + *i*.*c*. 1 x 10^5^ aZIKV (D14)	8	46	50.0	<0.001*
GL261 + Vax + *i*.*c*. Vax (D14)	8	n/a	75.0	<0.001*

Summary of descriptive statistics in second mouse tumor study. Data were from a single independent experiment.

^a^ Pairwise Log-Rank tests of each treatment group against untreated GL261 mice was corrected using Bonferroni method for multiple comparisons. *p* < 0.0166 is considered statistically significant.

**Fig 6:**
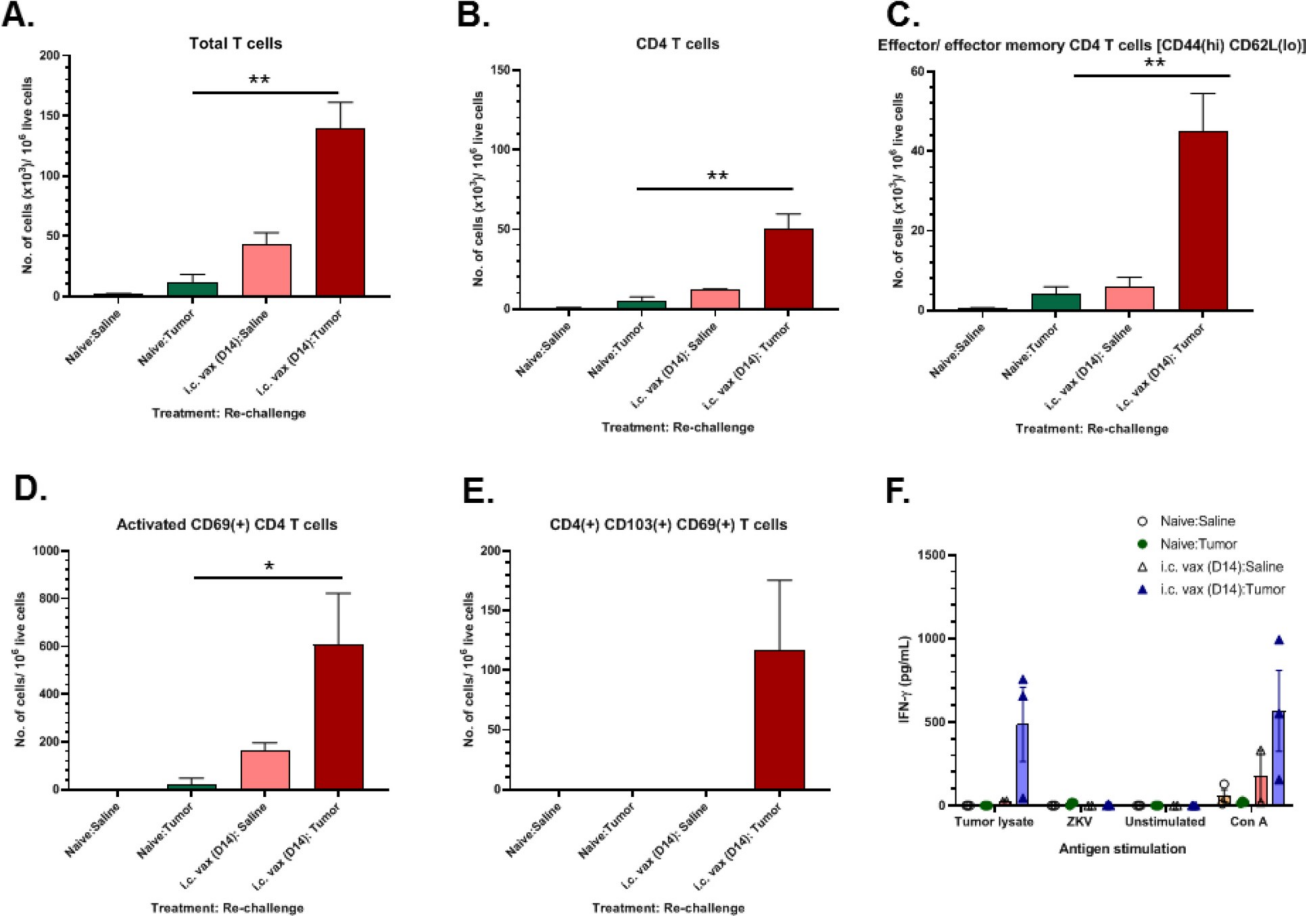
Phenotypes of infiltrating and resident immune cells and recall T-cell response in tumor rechallenged mice. Flow cytometry analysis of mononuclear cells within the brains of long-term surviving mice treated with *i*.*c*. vaccine in second mouse tumor study 7 days following tumor rechallenge (i.c. Vax: Tumor, *n* = 3), long-term survivors 7 days following *i*.*c*. injection of saline (i.c. Vax: Saline, *n* = 3), age-matched naïve-mice 7 days following *i*.*c*. tumor injection (Control: Tumor, *n* = 3), or age-matched naïve-mice 7 days following *i*.*c*. saline injection (Control: Saline, *n* = 3). Number of (A) CD3^+^ T-cells, (B) CD4^+^ T-cells, (C) CD4^+^ effector and effector memory T-cell, (D) activated CD69^+^ CD4^+^ T-cells, and (E) activated CD69^+^ CD103^+^ CD4^+^ memory resident T-cells. (F) ELISA quantification of IFNγ secretion by lymphocytes isolated from cervical lymph nodes after 72 h *ex vivo* stimulation with tumor lysate or ZIKV in long-term survivors or age matched controls following rechallenge with tumor or saline. Data was acquired from a single independent experiment. Data represented as mean ± SEM, (*p < 0.05; **p < 0.01; ***p < 0.001).

To investigate the immune response of mice treated with subcutaneous vaccination coupled with *i*.*c*. vaccines, long-term survivors were rechallenged with GL261 cells approximately 86 days following tumor induction. The brains and CLN of animals were removed 7 days following rechallenge. Flow cytometry analysis of mononuclear cells isolated from brain tissue revealed treated rechallenged mice significantly increased levels of total T-cells (CD3^+^) within the brain (Figs 6A and S2), including CD4^+^ T-cells (Fig 6B), effector and effector memory CD4^+^ T-cells (Fig 6C), and activated CD69^+^ CD4^+^ T-cells (Fig 6D), relative to naïve age-matched GL261-mice. Activated CD103^+^ resident memory T-cells within the brain were observed only in *i*.*c*. vaccinated mice rechallenged with tumor (Fig 6E). T-cells isolated from CLN were collected for functional assessment of antigen-specific IFN-γ production. Mice rechallenged with tumor following treatment with *i*.*c*. vaccination had increased levels of IFN-γ recall response when stimulated *ex vivo* with tumor lysate, but not when stimulated with ZIKV (Fig 6F). The results from this immunotherapy study further delineate the role of ZIKV as an adjuvant that can enhance tumor vaccines and is capable of improving OS to 75% in GL261-mice.

## Discussion

Virotherapy is an enticing approach for GBM as it exploits the propensity for viruses to infect and lyse tumor cells, leading to immune activation [17–20]. In the present study, we observed that ZIKV alone had no therapeutic effects as an oncolytic virus in a mouse or rat glioma model, in contrast to work by other groups [6, 8]. It is likely that the strain of ZIKV and animal model used can account for the differences in oncolytic activity *in vivo*. Zhu and colleagues treated immunocompetent glioma-bearing mice with a Dakar strain of ZIKV which was serially passaged through a *Rag1*^*-/-*^ mouse, thereby increasing its virulence, specifically in mice [21], while Chen and colleagues treated immunodeficient tumor-bearing mice with a live-attenuated Cambodian strain [22]. Although the number of reported ZIKV infection cases have significantly reduced following the Brazilian outbreak, ZIKV remains an active concern due to the neurotropism of the virus that can result in microcephaly in fetuses of infected pregnant women. Any attempt to move a ZIKV-based tumor therapy into the clinic must provide a thorough analysis of the product to ensure safety for the patients, clinicians, and caregivers.

While ZIKV was ineffective alone, combining virotherapy with other immunotherapy treatments is emerging as an effective preclinical paradigm for cancer treatment [23]. Recently, Zhu and colleagues demonstrated that ZIKV infection of glioma stem cells *ex vivo* elicits an immune response including increased expression of genes related to adaptive immunity, antigen presentation, interferon response, and Toll-like receptor signaling pathways [24]. Based on the potential for ZIKV to stimulate an immune response, we hypothesized that intratumoral ZIKV injections would penetrate the immunosuppressive tumor microenvironment and recruit T-cells generated by subcutaneous vaccinations to the tumor site to eliminate cancer cells. This was evident through the increase in OS in our rat tumor study and second mouse tumor study, where two-thirds of 9L-rats and half of GL261-mice treated with *i*.*c*. aZIKV survived long-term. The difference in OS between live ZIKV and aZIKV in mice and rats, however, is interesting and warrants further investigation. The administration of additional tumor antigen with ZIKV via *i*.*c*. vaccines produced an even greater OS, at 75%. We believe the enhanced OS in the *i*.*c*. aZIKV treated group and *i*.*c*. vaccine treated group is due to the altered treatment schedule in which we targeted *i*.*c*. injections to the reported peak or T-cell proliferation arising from subcutaneous vaccinations [16].

Successful outcomes following immunotherapies for the treatment of cancer have been linked to the generation of effector CD8^+^ and CD4^+^ T-cell responses, followed by the establishment of memory T-cell population capable of combatting re-emergence of the tumor [25]. In the current study, we observed an increase in the total number of T-cells, CD4^+^ T-cells, and memory T-cells following tumor rechallenge in treated GL261-mice surviving long-term. CD4^+^ T-cells are crucial in orchestrating antitumor immunity, with growing evidence highlighting the role of CD4^+^ T-cells in combatting the immunomodulatory effects of tumors [26]. In addition to the role of effector CD4^+^ T-cells, CD4^+^ memory T-cells are instrumental in protecting against future challenges and are the basis for successful vaccines [27]. Our data, in addition to supporting the role of CD4^+^ T-cells following treatment, highlight the importance of effector and effector memory CD4^+^ T-cells following glioma reoccurrence. By eliciting a CD4^+^ T-cell response and generating memory T-cells selectively responsive to tumor antigen, ZIKV-based immunotherapy offers a promising avenue for providing long-term protection against glioma.

## Supporting information

S1 Table Primers for qRT-PCR. (DOCX)

S1 Fig Flow cytometry analysis of immune cells in the brain following rechallenge of long-term survivors of first vaccine treatment study. Flow cytometry analysis of immune cells in the brain following rechallenge of long-term survivors of first vaccine treatment study. Mononuclear cells isolated from brain were analyzed by flow cytometry as described in materials and methods. (A). Flow cytometry gating strategy to analyze immune cells in the brain. Debris (SSC-A vs FSC-A) and doublets (SSC-H vs SSC-A) were excluded and discrimination of live and dead cells were determined based on Live/Dead fixable Aqua dead cell staining. Live cells were gated to identify dendritic cells [DCs; CD45(hi) CD11c(+)], myeloid cells [CD45(hi) CD11b(+)], lymphoid cells [CD45(hi) CD11b(-)], and microglia [CD45(int) CD11b(+)]. CD45(hi) CD11b(-) cells were sub-gated to identify CD19(+) B cells and CD3(+) T cells. Effector memory and memory CD4 T cells were identified as CD44(hi) CD62L(lo) cells and activated CD4 T cells were identified based on CD69 expression. (B) Representative flow cytometry plots showing expression of memory and activation markers in different groups of mice. Isotype specific antibodies were used to control for nonspecific antibody binding and to determine positive gating. T:V:T- long-term survivors 7 days following *i*.*c*. injection of tumor; T:V:S- long-term survivors 7 days following *i*.*c*. injection of saline; N:T- age-matched naïve-mice 7 days following *i*.*c*. tumor injection; N:S- age-matched naïve-mice 7 days following *i*.*c*. saline injection.
(TIF)

S2 Fig Flow cytometry gating strategy to analyze immune cells in the brain following rechallenge of long-term survivors of second vaccine treatment study. Flow cytometry gating strategy to analyze immune cells in the brain following rechallenge of long-term survivors of second vaccine treatment study. Mononuclear cells isolated from brain were analyzed by flowcytometry as described in materials and methods. Debris (SSC-A vs FSC-A) and doublets (SSC-H vs SSC-A) were excluded and live cells were gated to identify CD19(+) B cells and CD3(+) T cells. Memory CD4 T cells were identified as CD44(hi) CD62L(lo) cells, which are sub-gated based on CD103 expression as tissue resident memory T cells. Isotype specific antibodies were used to control for nonspecific antibody binding and to determine positive gating.
(TIF)

S1 File Raw data. Excel spreadsheet of raw data from mouse and rat survival studies, IFNγ secretion by lymphocytes, and CT values from qRT-PCR.
(XLSX)

S1 Raw images Raw images of PCR gel. Uncropped images of PCR gels related to Fig 1.
(TIFF)
