# Supplementary material for: Zika virus-based immunotherapy enhances long-term survival of rodents with brain tumors through upregulation of memory T-cells
Source: PLoS One. 2020 Oct 1;15(10):e0232858. doi: 10.1371/journal.pone.0232858 (PMC7529292; doi:10.1371/journal.pone.0232858)
Supplement: S1 Table — (DOCX) [file pone.0232858.s001.docx]

| **S1 Table. Primers for qRT-PCR** | |
| --- | --- |
| **Mouse** | |
| HPRT F | TCAGTCAACGGGGGACATAAA |
| HPRT R | GGGGCTGTACTGCTTAACCAG |
| MDA5 F | CTTGCTTCGAGAAGGGACTATT |
| MDA5 R | CTTCTCAGCAGCTCTCTTACAC |
| RIG-I F | GAGCCAGCGGAGATAACAATA |
| RIG-I R | CCCACGTACTCATAGAGAATGAC |
| TLR3 F | GTGCATCGGATTCTTGGTTTC |
| TLR3 R | GACCCAGTCTCTGTCTTTATGG |
| **Zika virus** | |
| ZKV-E F | TTGGTCATGATACTGCTGATTGC |
| ZKV-E R | CCTTCCACAAAGTCCCTATTGC |
| ZKV-NS2 F | TACTCACAGCTGTTGGCCTG |
| ZKV-NS2 R | CCCATGTGATGTCACCTGCT |
| ZKV-NS5 F | CAGCTGGCATCATGAAGAATC |
| ZKV-NS5 R | CACCTGTCCCATCTTTTTCTCC |
