# Supplementary figures and images for: Zika virus-based immunotherapy enhances long-term survival of rodents with brain tumors through upregulation of memory T-cells
Source: PLoS One. 2020 Oct 1;15(10):e0232858. doi: 10.1371/journal.pone.0232858 (PMC7529292; doi:10.1371/journal.pone.0232858)

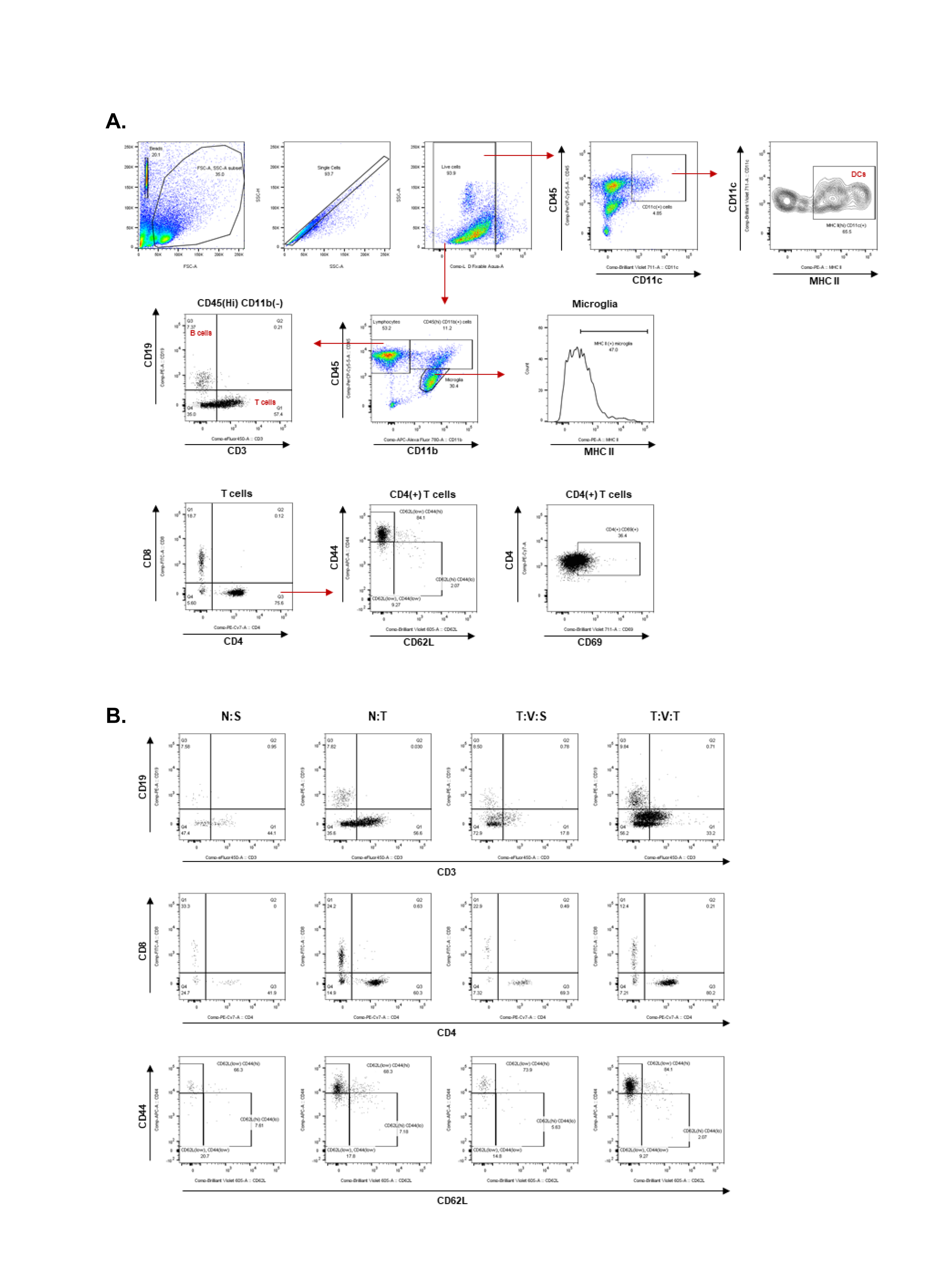

Supplement: S1 Fig — Flow cytometry analysis of immune cells in the brain following rechallenge of long-term survivors of first vaccine treatment study. Mononuclear cells isolated from brain were analyzed by flow cytometry as described in materials and methods. (A). Flow cytometry gating strategy to analyze immune cells in the brain. Debris (SSC-A vs FSC-A) and doublets (SSC-H vs SSC-A) were excluded and discrimination of live and dead cells were determined based on Live/Dead fixable Aqua dead cell staining. Live cells were gated to identify dendritic cells [DCs; CD45(hi) CD11c(+)], myeloid cells [CD45(hi) CD11b(+)], lymphoid cells [CD45(hi) CD11b(-)], and microglia [CD45(int) CD11b(+)]. CD45(hi) CD11b(-) cells were sub-gated to identify CD19(+) B cells and CD3(+) T cells. Effector memory and memory CD4 T cells were identified as CD44(hi) CD62L(lo) cells and activated CD4 T cells were identified based on CD69 expression. (B) Representative flow cytometry plots showing expression of memory and activation markers in different groups of mice. Isotype specific antibodies were used to control for nonspecific antibody binding and to determine positive gating. T:V:T- long-term survivors 7 days following i.c. injection of tumor; T:V:S- long-term survivors 7 days following i.c. injection of saline; N:T- age-matched naïve-mice 7 days following i.c. tumor injection; N:S- age-matched naïve-mice 7 days following i.c. saline injection. (TIF) [file pone.0232858.s002.tif]

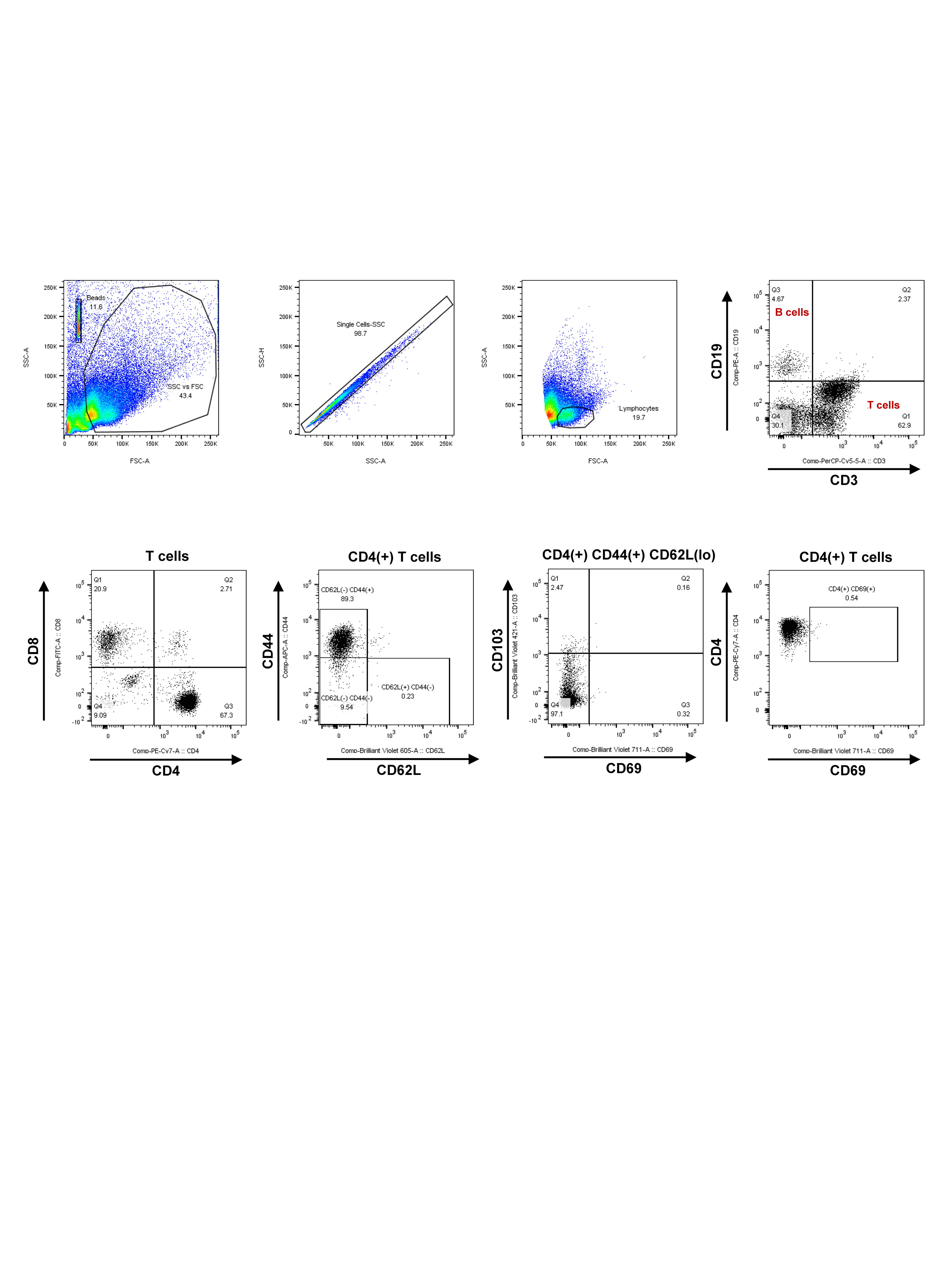

Supplement: S2 Fig — Flow cytometry gating strategy to analyze immune cells in the brain following rechallenge of long-term survivors of second vaccine treatment study. Mononuclear cells isolated from brain were analyzed by flowcytometry as described in materials and methods. Debris (SSC-A vs FSC-A) and doublets (SSC-H vs SSC-A) were excluded and live cells were gated to identify CD19(+) B cells and CD3(+) T cells. Memory CD4 T cells were identified as CD44(hi) CD62L(lo) cells, which are sub-gated based on CD103 expression as tissue resident memory T cells. Isotype specific antibodies were used to control for nonspecific antibody binding and to determine positive gating. (TIF) [file pone.0232858.s003.tif]
